# Construction of a nutritional assessment tool for the pediatric cancer population (ANPEDCancer): content validation using the Delphi method

**DOI:** 10.1590/1984-0462/2023/41/2021126

**Published:** 2022-09-09

**Authors:** Danúbia da Cunha Antunes Saraiva, Renata Brum Martucci, Gina Torres Rego Monteiro

**Affiliations:** a Instituto Nacional de Câncer José Alencar Gomes da Silva, Rio de Janeiro, RJ, Brazil.; b Fundação Oswaldo Cruz, Escola Nacional de Saúde Pública Sérgio Arouca, Rio de Janeiro, RJ, Brazil.

**Keywords:** Nutrition assessment, Pediatrics, Delphi technique, Consensus, Validation study, Avaliação nutricional, Pediatria, Técnica Delfos, Consenso, Estudo de validação

## Abstract

**Objective:**

To develop and establish content validation of a nutritional assessment tool for pediatric cancer patients using the Delphi method.

**Methods:**

A literature review was performed and the nutritional assessment in pediatrics and cancer construct was discussed with experts. Forty-six nutrition experts from Brazil with experience in oncology participated in the panel. Rounds were held to discuss and judge the items that should be included in this tool. With the aid of an algorithm, it was possible to simultaneously consider the adequacy, relevance and measurement of the items. The consensus was reached with an agreement equal to or greater than 80% among the experts.

**Results:**

From the 7 domains suggested by the literature, the first round generated 81 items, which were assessed for adequacy, relevance and the possibility of being measured in the routine of nutritional assessment, by specialists in the following two rounds. The percentage of specialists who responded to each round was high (above 90%) and the dropout rate between the stages was relatively low. After applying the algorithm, the ANPEDCancer tool had 36 items considered appropriate by specialists from the five different geographical regions of Brazil.

**Conclusions:**

The Delphi method allowed the construction and content validation of the nutritional assessment tool for children and adolescents with cancer, providing the first stage for use in the Brazilian hospital setting.

## RESUMO

**Objetivo:**

Desenvolver e estabelecer a construção, a validação de conteúdo e a elaboração de um plano de ação para cada diagnóstico nutricional de um instrumento de avaliação nutricional para pacientes pediátricos com câncer por meio do método Delphi.

**Métodos:**

Foi realizada uma revisão da literatura e discutido com especialistas o constructo avaliação nutricional em pediatria em câncer. Participaram do painel 46 especialistas nutricionistas do Brasil com experiência em oncologia. Rodadas ocorreram, para discutir e julgar os itens que deveriam constar do instrumento. Com o auxílio de um algoritmo, foi possível ponderar simultaneamente a adequação, relevância e mensuração dos itens. O consenso foi atingido com concordância igual ou superior a 80% entre os especialistas.

**Resultados:**

Com base nos sete domínios sugeridos pela literatura científica, a primeira rodada gerou 81 itens, que foram avaliados quanto à adequação, relevância e possibilidade de ser medido na rotina de avaliação nutricional pelos especialistas nas duas rodadas seguintes. O percentual de especialistas que responderam a cada rodada foi alto (acima de 90%), e a desistência entre as etapas, relativamente baixa. Após aplicação do algoritmo, o instrumento ANPEDCancer contou com 36 itens considerados apropriados pelos especialistas das cinco distintas regiões geográficas do Brasil.

**Conclusões:**

O método Delphi permitiu a construção e validação de conteúdo do instrumento de avaliação nutricional para crianças e adolescentes com câncer, fornecendo uma primeira versão a ser aplicada e avaliada no cenário hospitalar brasileiro.

## INTRODUCTION

Adequate nutritional status is essential in children and adolescents with cancer to improve short-term clinical outcomes and long-term health. The etiology of malnutrition in childhood cancer is multifactorial, with a complex interaction between treatment side effects, energy metabolism, and other factors. The prevalence of malnutrition in pediatric cancer patients can be three times higher than in the general Brazilian population in the same age group,^1^ and this reality can be worse, especially in low- and middle-income regions. It is known that malnutrition is associated with greater morbidity and mortality.^2^ Therefore, it is essential that there are nutritional instruments and procedures that offer a more effective approach to pediatric cancer patients.

Nowadays, only the SCAN instrument (nutritional screening for childhood cancer) assesses the nutritional risk in children and adolescents with cancer.^3^ The disadvantage of using this tool is that, after identifying the nutritional risk, it is necessary to refer the patient to the dietitian for a complete nutritional assessment, which, in many cases, boils down to complementing with anthropometry, which, for some authors, is not considered sufficient in isolation to reflect the nutritional status.^4^

The Delphi method has been widely used in scientific research for the development of quality indicators, guidelines, and the construction of instruments in health services.^5–8^ It enables the survey of items of interest based on the experience of specialists and allows a large number of individuals in different locations and areas of expertise can be included anonymously, thus avoiding consensus domination by one or a few experts.^5,8–10^

The validation of an instrument verifies that it measures what it is intended to measure. Content validation consists of exploring the construct of interest based on an extensive and rigorous review of the scientific literature, experiences, and observations of the researcher himself, involving a discussion with a group of experts.^11^

The objective of this study was to describe the methodological steps of the construction and content validation, through the Delphi technique, of the new nutritional assessment instrument ANPEDCancer (Nutritional Assessment of Pediatric Cancer Patients). The ANPEDCancer is a structured instrument, through its domains, created by dietitians with the purpose of comprehensively assessing the nutritional status of children and adolescents hospitalized with cancer.

## METHOD

This methodological study was carried out in two stages. The first was the preparation phase, with the specification of the “nutritional status” construct, identifying the relevant domains of nutritional assessment in pediatrics and oncology through an extensive review of the scientific literature on the PubMed database with the following Medical Subject descriptors Headings (MeSH): nutritional status, nutrition assessment, pediatrics, neoplasms, diagnosis, and screening.

In the second stage, there were rounds to discuss the items relevant to the construction of the instrument, using the Delphi method with the adaptations pointed out by the studies by Mokkink et al.^9^ and Magarinos-Torres et al..^12^ The Delphi method is a structured process that uses a series of questionnaires or rounds to collect information, interspersed with controlled feedback. Rounds occur until group consensus is reached. The use of the internet made it possible to include specialists from various regions of the country, contributing to a potential diversity of knowledge on the topic in focus.^7,13,14^ In this work, communication with experts was carried out from October 2018 to January 2019, using the SurveyMonkey online survey system.

First, in order to invite dietitians with expertise in pediatric oncology, a strategy was needed to locate them. To this end, units of High Complexity Care Centers in Oncology (Cacons/Unacons) accredited, with pediatric oncology service, of the Federative Units of Brazil were identified, and invitations were sent to dietitians who worked in them to participate in the research. The invitation explained that the participation was personal and that, therefore, the professional did not represent the institution in which they worked.

The criteria to be part of the panel were: knowledge of nutritional assessment in pediatrics and oncology and professional experience (minimum of two years). Fifty-four experts were invited, of which 48 agreed to participate in the research. The panel was designed with the aim of including representatives from the five regions of the country, to allow the construction of an instrument capable of being used in different regions of Brazil and to capture the professional practice of dietitians, making it possible to reach, in part, the diversity of the Brazilian pediatric population.

In order to conduct the Delphi method, the authors previously decided to follow the practical guidelines,^5^ in order to guarantee the legitimacy of the process. The composition of the expert panel was in accordance with the purpose of the study. The structuring of the questionnaires for each round, with open and closed questions, was carried out with the aim of encouraging the contribution and suggestion of pertinent items and considerations, according to the scope of the research.

The system for sending questionnaires over the internet, the choice of method to inform participants of the results of previous rounds (questionnaires), measures to minimize losses to follow-up of participants, and attention to the time required for participants to respond in each round were carried out to optimize the entire conduction of the Delphi method.

After reviewing published scientific studies on the assessment of nutritional status in pediatrics and oncology, a questionnaire, called the First Working Document, was developed with the relevant domains in the nutritional assessment of pediatric cancer patients, and based on this document, the rounds of the study were initiated.

The First Working Document was sent to the specialists, with 15 questions, between open and closed, and their opinion was requested in each of the suggested domains, stimulating a brainstorming, in order to obtain the items to compose the final instrument. The first question focused on whether the model contemplated all important domains in the nutritional assessment of pediatric cancer patients and encouraged the specialist to include any item (or consideration) that had not been covered. From the second to the 12^th^ question, the specialist would have to assess whether the items included were relevant, adequate, and should be used in an assessment instrument. Questions 13 and 14 asked about the criteria for diagnosis and the action plan for each nutritional diagnosis found.

An open question asked the specialists, for each nutritional diagnosis, which measures in nutritional assistance should be taken, that is, what was the action plan for each “well-nourished/eutrophic” patient, with “nutritional risk”, “malnourished”, and with “risk of/or overweight/obesity”. The last question reinforced the possibility of adding some item or consideration to the instrument.

In this first stage, the deadline for returning the questionnaire was 12 calendar days. In all, 46 experts returned with their contributions to the first stage. All responses and observations were compiled and synthesized to deepen the discussion in the next step.

The Second Working Document was organized with the consolidated contributions of all 46 experts who responded to the First Working Document. Thus, each specialist became aware of the synthesis of the group’s contribution to each item suggested by the previous step and was able to judge, among them, those suitable for a nutritional assessment instrument, subject to measurement in clinical practice and relevant to be measured in different contexts of the Brazilian hospital nutritional assistance. The decision criterion for the items, both to assess adequacy and to be measurable, was yes and no. Item relevance was measured on a scale from 0 to 2, where: 0 is irrelevant, 1 is slightly relevant, and 2 is relevant. At this stage, the deadline for returning the questionnaire was eight calendar days.

After receiving the experts’ considerations, weights were attributed to the answers, which were analyzed based on an algorithm based on a decision tree, with the aim of simultaneously weighing the three aspects addressed. The algorithm was built assuming the greatest weight to suitability, followed by relevance and, finally, being measurable.

The answers provided by 42 experts in the second round were analyzed and, based on them, the Third Working Document was prepared. This step aimed to review the items that did not present consensus (<80%). Along with the consolidated, each specialist received their own answers for each item in another document, by e-mail, and was instructed that, when answering the questionnaire, they could maintain or modify their previous answer. In this last stage, the return period was seven calendar days. All the answers provided were systematized and analyzed with the same algorithm.

The results on adequacy and on being measurable and relevant were analyzed according to descriptive statistics (absolute and percentage frequencies) in the statistical program Statistical Package for the Social Sciences (SPSS), version 22. The items considered in agreement were those that reached at least 80% of the answers of the specialists, being considered of consensus. Non-relevant items were excluded from the instrument.

This work was approved by the Research Ethics Committees of the Sergio Arouca National School of Public Health, the Oswaldo Cruz Foundation (opinion number 2,557,807) and the José Alencar Gomes da Silva National Cancer Institute (*Instituto Nacional de Câncer José Alencar Gomes da Silva* – INCA) (opinion number 2,601,409), under the Certificate of Presentation of Ethical Assessment (*Certificado de Apresentação de Apreciação Ética* – CAAE) 73737317.2.0000.5240.

## RESULTS

A total of 46 dietitians from different regions of Brazil participated in the expert panel, of which 39 (84.8%) responded to all stages. The study had, therefore, a loss of follow-up of 15.2%, with 8.7% (n=42/46 participants) from the first to the second round and 7.1% (n=39/42 participants) from this to the third round. In the South, Southeast, and Midwest regions, all states were represented in the three stages of the process. The distribution of specialists at the end of the process was 43.5% in the Southeast Region, 18% in the Northeast and 12.8% in each of the other regions (Table 1).

**Table 1:** Characteristics of Delphi method experts, 2018.

			n (%)	
Gender				
	Female		45	(97.8)
	Male		1	(2.2)
Academic degree				
	PhD		4	(8.7)
	Master		11	(23.9)
	Specialization		30	(65.2)
	Graduation		1	(2.2)
Years of experience in pediatric oncology, median (min.-max.)			6	(2–20)
State of professional activity		1^st^ Round	2^nd^ Round	3^rd^ Round
Midwest region		5 (10.9)	5 (11.9)	5 (12.8)
	Federal District	2	2	2
	Goiás	1	1	1
	Mato Grosso	1	1	1
	Mato Grosso do Sul	1	1	1
Northeast region		9 (19.6)	8 (19)	7 (18)
	Bahia	3	3	3
	Maranhão	1	1	1
	Pernambuco	2	2	2
	Rio Grande do Norte	2	1	1
	Sergipe	1	1	0
North region		6 (13)	6 (14.3)	5 (12.8)
	Acre	1	1	1
	Amazonas	1	1	1
	Pará	3	3	3
	Roraima	1	1	0
Southeast region		20 (43.5)	17 (40.5)	17 (43.6)
	Espírito Santo	1	1	1
	Minas Gerais	2	2	2
	Rio de Janeiro	7	6	6
	São Paulo	10	8	8
South region		6 (13)	6 (14.3)	5 (12.8)
	Paraná	2	2	2
	Rio Grande do Sul	1	1	1
	Santa Catarina	3	3	2
Total		**46**	**42**	**39**

min.: minimum; max.: maximum.

According to the responses in the First Working Document, 52.2% (n=24/46) of the experts considered that the model covered all important domains in the nutritional assessment of pediatric cancer patients:

- Anthropometric assessment by Body Mass Index for Age (BMI/A) or Mid-Upper Arm Circumference (MUAC).
- Adequacy of body weight.
- Adequacy of nutritional intake.
- Gastrointestinal symptoms.
- Clinical/oncological condition.
- Physical examination/clinical observation.
- Plan of action (nutritional diagnosis that guides the conduct to be performed).

The domain anthropometric assessment by BMI/A or MUAC received several suggestions for the inclusion of items, with consensus on some of them only obtained after the third round. The experts pointed out that using only BMI/A or MUAC was not adequate to assess the nutritional status of pediatric cancer patients, and it was necessary to include all anthropometric indicators (allowing the use of the most appropriate one at the time of evaluation) and complement them with MUAC.

The evaluation of the physical examination domain, carried out with the question “Should the dietitian perform the physical examination/clinical observation of patients as part of the nutritional assessment?”, obtained agreement from all the specialists. They considered it important to assess: loss of visible muscle mass (98%, n=45); visible subcutaneous fat loss (98%, n=45); presence of edema (98%, n=45); ascites (93%, n=43); anasarca (93%, n=43); and skin changes (91%, n=42). It was also suggested to include the assessment of nine other items: examination of the oral cavity; nails; hair/absence of hair; presence of decubitus pressure on the skin; observation of the abdomen; presence of bulky mass; presence of skin lesions; presence of skin pallor; and jaundice.

The action plan for each nutritional diagnosis was built with the contributions of all specialists, after the first round, through the compilation of responses and aggregation of similar behaviors, and was judged in the second round. The reassessment times proposed by the experts, on average, for each nutritional diagnosis of ANPEDCancer were 12 days for well-nourished patients, 6.2 days for those at nutritional risk, 6.8 days for malnourished ones, and 8.8 days for individuals at risk of/or overweight/obesity.

After analyzing and synthesizing the responses from the first round, 81 items were included for the second round. As the objective of this methodological step was to assess the adequacy, measurement, and relevance of the items, it was decided by the researchers to include all those that had been suggested in the first round to be judged by all the experts.

In the second round, an expert proposed the inclusion of a new item for the action plan domain: “*The reassessment routine must be based on the current nutritional diagnosis*”. Although this item was not evaluated in the second round by all the specialists, it was considered very relevant by the researchers and included to be evaluated in the third round.

The predicted minimum percentage of agreement among experts regarding adequacy (80%) was not reached by 22 items in the second round, and the items were then re-evaluated in the third round. Only six of them presented a percentage higher than 80%, being included for evaluation in the decision algorithm, as well as the other items previously answered.

**Figure 1:**
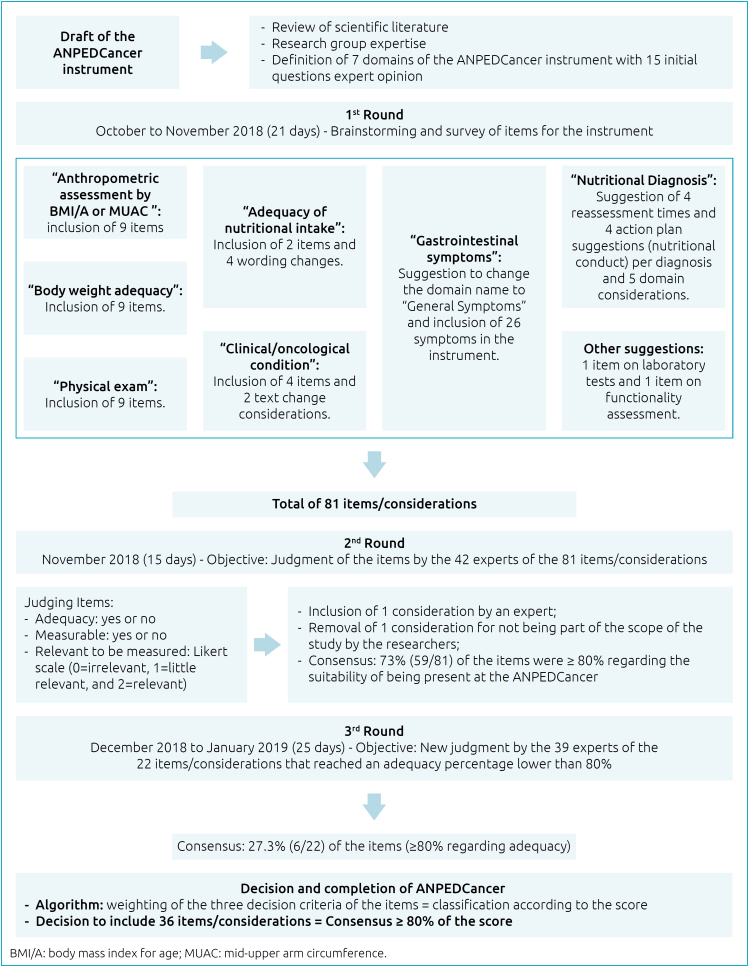
Summary of the development of the nutritional assessment instrument for hospitalized pediatric cancer patients (ANPEDCancer) and validation of its content by the Delphi method.

The algorithm was applied to all 81 items evaluated by the experts, and 36 obtained a maximum score of 100 points, being classified as adequate, relevant, and capable of being measured in the dietitian’s care practice, as can be seen in Table 2. It should be noted that there was agreement greater than 92% on the action plan to be taken according to the nutritional diagnosis in all four suggested outcomes. The summary of the development of the ANPEDCancer instrument can be seen in Figure 1.

**Table 2:** Results with the highest agreement rate for the three criteria addressed (adequate, measurable, and relevant to be measured) according to the decision algorithm.

Domains	Items	Consensus (%)
Anthropometric assessment	Include all anthropometric indicators	80.9
	Use anthropometric indices and MUAC	82.0
Adequacy of food intake	Include: food preferences, allergies, restrictions	88.1
	Change in the text: “did not present a reduction in food intake or maintained a good dietary pattern”	80.9
	Change in the text: “in the last days”	80.9
	Use a scale to quantify food intake	80.9
Body weight adequacy	Use weight loss percentage calculation	92.9
	Add: presence of edema	95.2
	Assessment of weight evolution in patients regardless of age	83.3
Gastrointestinal symptoms	Inappetence/hyporexia	95.2
	Anorexia	88.1
	Diarrhea	97.6
	Constipation	90.5
	Nausea/urge to vomit	97.6
	Vomiting/emesis	97.6
	Mucositis in GIT	100
	Dysgeusia/altered taste	92.9
	Odynophagia	92.9
	Dysphagia	100
	Xerostomia	90.5
	Abdominal pain/discomfort	90.5
	Oral cavity injury	95.2
	Gastroesophageal reflux	80.95
	Enterocolitis	80.95
	Abdominal distension	88.1
Clinical/oncological condition	Include in high nutritional risk: patient in a pediatric intensive care unit	88.1
Physical exam	Assess oral cavity (mucositis, moniliasis)	83.3
	Assess abdomen (flaccid, globular, tense, distended)	92.9
	Assess the presence of a bulky mass (abdominal, lower and upper limbs, neck)	80.9
Nutritional diagnosis/plan of action	Plan of action – eutrophic	95.2
	Plan of action– nutritional risk	97.6
	Plan of action – malnourished	95.2
	Plan of action – risk of/or overweight/obesity	92.9
	Reassessment: nutritional risk (mean 6.2 days)	89.7
	Reassessment: malnourished (mean 6.8 days)	84.6
	Routine assessment should be based on current nutritional diagnosis	89.7

MUAC: mid-upper arm circumference; GIT: gastrointestinal tract.

Regarding the period for returning the questionnaires by the experts at each round, there was some delay in all stages. To avoid further delay, a reminder had been programmed and effectively sent by e-mail, before the deadline. Even with this behavior, the first round was completed in 21 days, the second in 15 days, and the third in 25 days.

**Figure 2:**
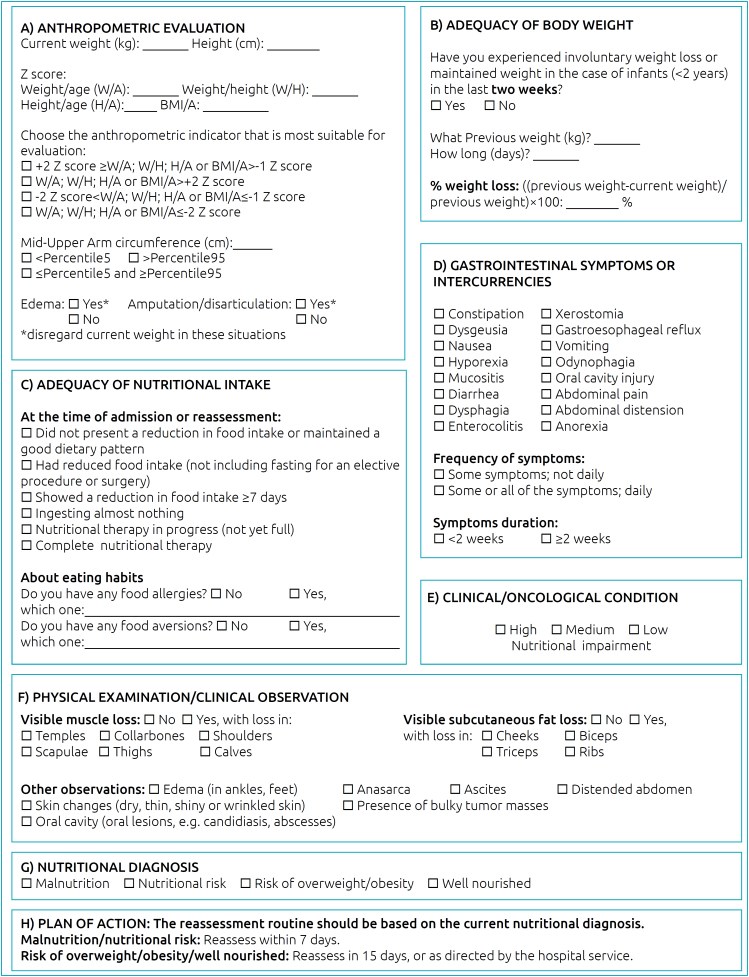
ANPEDCancer instrument for nutritional assessment of hospitalized pediatric cancer patients.

Finally, Figure 2 presents the ANPEDCancer, an instrument for nutritional assessment with an action plan for nutritional care for pediatric cancer patients, agreed by experts and discussed among the researchers regarding the best presentation and layout of the version to be used by dietitians in the care practice. The instrument was separated into domains: anthropometric assessment, adequacy of body weight, adequacy of nutritional intake, gastrointestinal symptoms or complications, clinical/oncological condition (detailed in Chart 1), physical examination/clinical observation and, finally, the nutritional diagnosis with the respective action plan for conduct and nutritional reassessment (Chart 2).

**Chart 1:** Construction by experts of clinical/oncological condition examples for use in the ANPEDCancer tool.

Clinical/Oncological Condition		
High nutritional impairment	Medium nutritional impairment	Low nutritional impairment
□ Medulloblastoma □ Neuroblastoma □ Wilms tumor □ Osteosarcoma □ Ewing sarcoma □ Rhabdomyosarcoma □ Acute myeloid leukemia □ Hodgkin lymphoma □ Head and neck tumors □ Diencephalic and Other CNS tumors □ Irradiation of the gastrointestinal tract □ Bone marrow transplantation □ Leukemia in relapse situations and high-risk group (protocol) □ Extensive abdominal surgery □ Presence of fistulas □ Frequent cycles of chemotherapy (interval ≤ 3 weeks) □ Highly emetogenic chemotherapeutics (e.g.: cisplatin, Cyclophosphamide (CTX), Methotrexate (MTX)) □ Gastrointestinal postoperative period (< 4 weeks) □ Organ failure (kidney, liver, lung, heart) □ Clinical conditions (HIV, colitis, pancreatitis) □ Metabolic abnormalities (acidosis, alkalosis, hypoglycemia, hyperglycemia) □ Infants (< 2 years) □ Patients in severe conditions (ICU)	□ Good prognosis of acute lymphocytic leukemia (Low Risk according to protocol) □ Oncological diseases in remission or during maintenance treatment; □ Chemotherapy with corticosteroids (such as prednisone; methylprednisone, dexamethasone); □ Non-metastatic solid tumors (which are not listed in the High-risk group) □ Fever (> 37.5°C, for 2 consecutive days)	□ Absence of fever in the last 48h* □ Absence of use of corticosteroids* *provided it does not meet any of the above conditions

CNS: central nervous system; HIV: human immunodeficiency virus; ICU: intensive care unit.

**Chart 2:** Nutritional action plan for each diagnosis of the ANPEDCancer tool prepared by the experts using the Delphi Method.

Nutritional diagnosis	Plan of action
Malnutrition	Reassess within 7 days. Optimize oral nutritional supplementation and/or indication of an alternative route for nutritional therapy. Regain nutritional status through adequacy of nutritional needs and symptom management. Nutritional education. Carry out individualized nutritional planning, considering the reported changes and daily monitoring. Immediate nutritional supplementation, offer tube feeding (nasoenteral or gastrostomy) if intake is <60% of what was planned, for 3 consecutive days.
Nutritional risk	Reassess within 7 days. Nutritional therapy best suited to the case (oral or enteral). Nutritional guidance and education. Adequacy of diet according to habits with the inclusion of more caloric foods (with good nutritional value). Regain nutritional status by adjusting nutritional needs and managing symptoms. Nutritional supplementation if intake < 75% of what was planned, propose tube feeding if intake is < 60% of what was planned.
Risk of overweight/obesity	Reassess within 15 days (or as directed by the hospital service). Assess and adjust food intake, as well as change the habits necessary for healthy eating. Evaluate the use of corticosteroids and use strategies to improve muscle mass. Make healthier adaptations within the patient’s eating habits. Do food reeducation. Maintain nutritional monitoring, verifying food acceptance, organizing a healthy eating plan in order to avoid further weight gain.
Well nourished	Reassess within 15 days (or as directed by the hospital service). Nutritional guidance on healthy eating and food safety (good hygiene practices, food handling). Qualitative and quantitative assessment of food intake, monitoring of the current therapeutic regimen. Monitor in order to maintain nutritional status. Monitoring of symptoms and nutritional follow-up.

## DISCUSSION

To our knowledge, this is the first study to develop a nutritional assessment instrument for children and adolescents with cancer, with its content validated by experts from all over Brazil using the Delphi methodology. The ANPEDCancer instrument includes seven domains, which, at the end of its application, allow classifying the nutritional status and directing an action plan, that is, it guides nutritional behavior.

Based on the expertise of the specialist professionals who participated in the process, ANPEDCancer complies with the international nutritional assessment recommendations of the American Society for Parenteral and Enteral Nutrition (ASPEN)^15^ and the European Society for Clinical Nutrition and Metabolism (ESPEN),^16^ as well as the Brazilian Society of Pediatrics,^17^ in identifying nutritional status and ensuring continuous monitoring of nutritional status. It is a promising instrument for the identification and monitoring of the health conditions of pediatric cancer patients by professionals in the field.

The number and representativeness of experts are strengths that affect the potential of ideas in Delphi. The larger the sample size, the greater the generation of data.^12^ Although there is no consensus on the number of specialists needed to use the Delphi method, it is important to consider losses to follow-up and develop strategies to keep specialists committed to all phases of the project.^5,8,18^ In this study, the loss was not high, when compared to others that present losses greater than 25%.^12,13,18^

Although it was not possible to have a representative from each state, as initially desired, the present study was able to include professionals from the five regions of Brazil, who work directly with pediatric cancer patients, making it possible to contemplate the country’s regional diversity in the construction of the instrument. The predominance of specialists in the Southeast region can be partly explained by the greater number of health units for pediatric oncology care in the region.^19^

The nutritional assessment domain had a greater suggestion of adding items, and only approximately half of the experts agreed with the model without modifications. One possible explanation is that dietitians already have consolidated knowledge that nutritional assessment needs to be performed based on all anthropometric parameters. In fact, the greater the number of parameters, the more reliable the assessment of nutritional status will be,^20–23^ however the purposes of this instrument are to present the fewest number of questions and achieve the most accurate result possible, so that it is feasible to be used in the practice, in different regions and resources (personnel and material) in hospitals, without losing the technical quality of evaluation.

Physical examination as part of the instrument showed high agreement among the experts in the study. Professional expertise and good clinical judgment are essential to perform a good physical examination on the patient. It is important for the dietitian to visually inspect the muscle and fat compartments in a nutritional assessment. In many situations, such as the presence of diseases or frailties of the patient that make the anthropometric measurements typically used impossible, the physical examination will be the guide for the nutritional diagnosis.^15,20^

The consensus process, in this work, took place in the second and third rounds by the experts. The high agreement obtained in all stages of the method confers legitimacy to the process.^8,14,24^ Another important point in the accuracy of the study is to ensure compliance with the guidelines regarding the conduct of the method, such as: adequacy of the composition of the expert panel according to the objective of the study, formulation of questionnaires for data collection, prior definition of the consensus criterion and time to stop the process (number of rounds), losses to follow-up and the duration of the study.^5,10^ All these guidelines were fulfilled.

The present study presented delays in the rounds, as observed in other studies.^12,13,25^ The Delphi process can be long, each round can take time to complete, and therefore there is a need to monitor non-respondents and assess the time needed to properly analyze the results, as well as prepare feedback for the next round.^5^

This study had some limitations. The first is not having all the states of Brazil represented, despite the effort expended in this regard. Some were not covered because they did not have Cacon/Unacon with pediatric oncology, especially in the North Region. The second was the lack of availability of some specialists to participate in all stages, but this limitation was minimized by the low percentage of loss, especially when compared to other studies on the subject. The possibility of unintentional influence on the conduct of the consensus process is another potential limitation. To try to avoid it, all the suggestions were accepted and exposed to the specialists in the next round, so that their importance could be analyzed, even if this implied a long questionnaire for judgment.

On the other hand, the present study presented advantages, the main one being the fact that the process was conducted completely remotely, making it possible to gather the opinion of qualified professionals from different geographic locations in Brazil, which would not be possible in person. This process eliminated the potential bias of influence of one expert on the others, as can occur in face-to-face meetings, and also allowed the number of steps that were necessary to be carried out until reaching the desired consensus. Another strong point of the study was the flexibility that each professional had to respond in the time and schedules available, which allowed for more consistent responses.

Finally, this study presented the construction and content validation of the ANPEDCancer, a nutritional assessment tool to be used in hospitalized pediatric cancer patients. It counted on the contributions of dietitians from the five regions of Brazil, encompassing the experience and practical and scientific experience for its use in the context of Brazilian hospitals. It is noteworthy that content validation is the first step for this tool to be available to be used and to be evaluated in the Brazilian hospital environment. The continuity of studies for criterion and construct validation and to estimate their reliability, analyzing internal consistency, equivalence (interobserver agreement) and stability (through test-retest), is a future and important proposal for its wide use in the Brazilian scenario, in different populations of children and adolescents with cancer.
